# Crystal structure of tris­(ethyl­enedi­ammonium) hexasulfatopraseodymium(III) hexa­hydrate

**DOI:** 10.1107/S1600536814020704

**Published:** 2014-09-20

**Authors:** Peter Held

**Affiliations:** a Institut für Kristallographie, Universität zu Köln, Greinstrasse 6, D-50939 Köln, Germany

**Keywords:** crystal structure, praseodymium, ethyl­enedi­ammonium, hydrogen bonds

## Abstract

In the title salt, (C_2_H_10_N_2_)_3_[Pr_2_(SO_4_)_6_]·6H_2_O, the Pr^III^ cation is surrounded ninefold by five sulfate groups (two monodentate and three chelating) and by one water mol­ecule [range of Pr—O bond lengths 2.383 (3) to 2.582 (3) Å]. The [Pr(SO_4_)_5_(H_2_O)] groups are arranged in sheets parallel to (010). Two crystal water mol­ecules and two ethyl­enedi­ammonium cations (one with point group symmetry -1) connect the sheets *via* O—H⋯O and N—H⋯O hydrogen bonds from weak up to medium strength into a three-dimensional framework structure.

## Abstract 2

The Pr^III^ cation is surrounded ninefold by five sulfate groups (two monodentate and three chelating) and by one water mol­ecule. The [Pr(SO_4_)_5_(H_2_O)] groups are arranged in sheets parallel to (010); two crystal water mol­ecules and two ethyl­enedi­ammonium cations connect the sheets *via* O—H⋯O and N—H⋯O hydrogen bonds into a three-dimensional framework structure.

## Chemical context   

In the course of a systematic search for new ‘double salts’ of simple secondary amines and mono- or divalent cations of various inorganic acids, the structures of (C_2_H_10_N_2_)[Li_2_(SO_4_)_2_] and (C_2_H_8_N)[Cu(HSO_4_)(SO_4_)(H_2_O)_4_] have been described previously (Held, 2003 ▶, 2014 ▶). In continuation of these studies, lithium was replaced by trivalent praseodymium, yielding crystals of the title compound with composition (C_2_H_10_N_2_)_3_[Pr_2_(SO_4_)_6_]·6H_2_O.
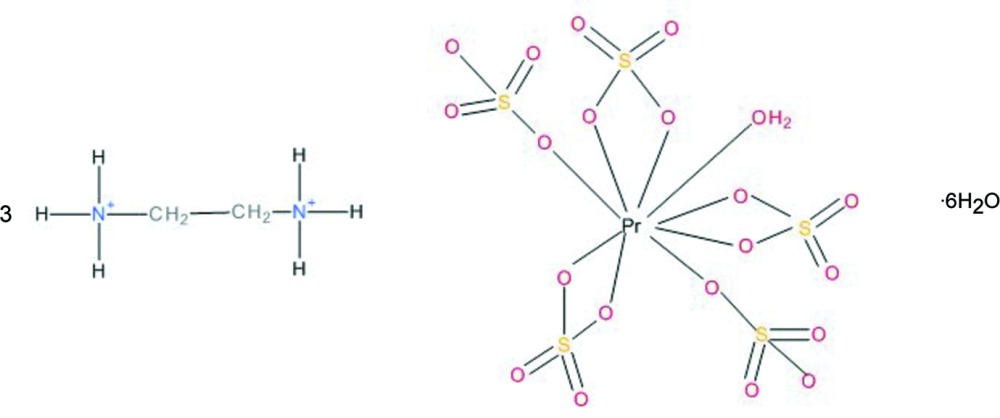


## Structural commentary   

**Figure 1:**
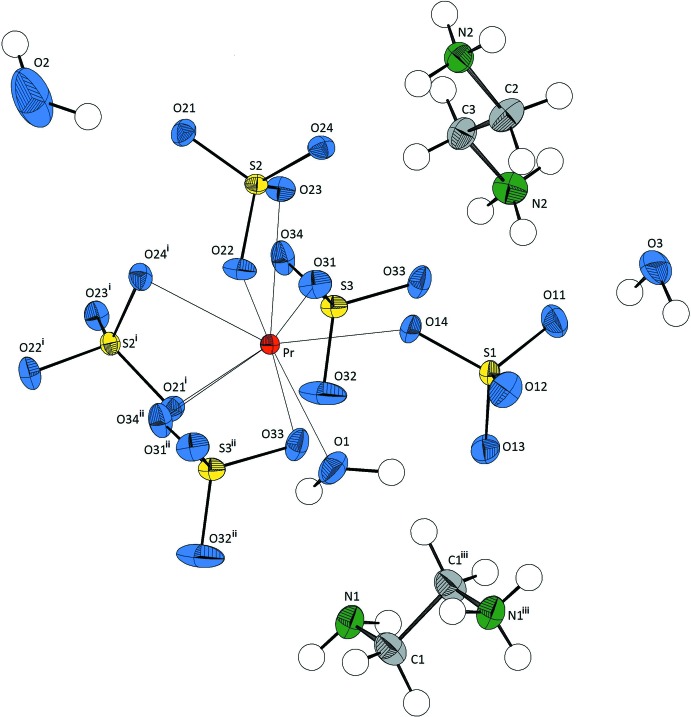
The mol­ecular entities in the structure of the title compound, showing the atom-numbering scheme. Displacement ellipsoids are drawn at the 50% probability level. [Symmetry codes: (i) *x*, −*y* + 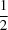, *z* + 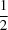; (ii) *x* + 1, *y*, *z*; (iii) −*x*, −*y*, −*z* + 1].

The asymmetric unit of the title compound contains three (SO_4_)^2−^ anions, one and a half [NH_2_(CH_3_)]^2+^ cations (the other half being generated by inversion symmetry), one Pr^3+^ cation as well as three water mol­ecules (Fig. 1 ▶). The Pr^3+^ cation is surrounded by nine O atoms from five sulfate groups, two of which are monodentately bonding and three chelating, and of one water mol­ecule. The averaged Pr—O distance in the resulting distorted monocapped square-anti­prism, [Pr(SO_4_)_5_(H_2_O)], is 2.52 (7) Å. Praseodymium reaches an overall bond-valence sum (Brown & Altermatt, 1985 ▶) of 3.23 valence units. The S—O distances are nearly equal [average distance 1.479 (13) Å], however, the O—S—O angles vary [average bond angle 109.48 (2.05)°] clearly. One sulfate group (S2) inter­connects two [PrO_9_] polyhedra *via* two common edges parallel to [001], while another sulfate group (S3) connects *via* a common edge and a common vertex parallel to [100], leading to the formation of sheets parallel to (010).

## Supra­molecular features   

**Figure 2:**
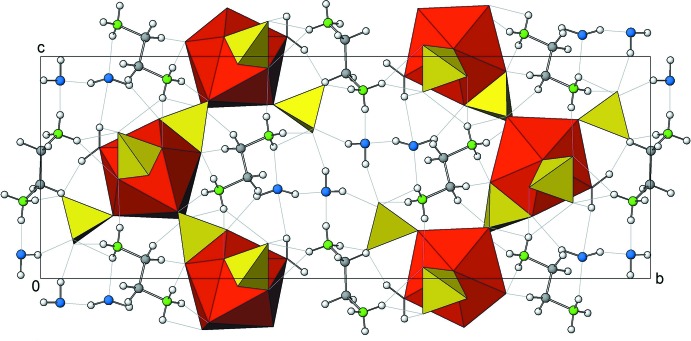
(100)-projection of the crystal structure of the title compound. Hydrogen bonds are shown as light-grey dashed lines. Colour scheme: (SO_4_) tetra­hedra (yellow), monocapped anti­prism [PrO_9_] (red), O (blue), N (green), C (grey), H (white).

Hydrogen bonds of medium strength involving water mol­ecules as donor groups and O atoms of the sulfate anions as acceptor groups inter­connect neighbouring [Pr(SO_4_)_5_(H_2_O)] units. Together with relatively weaker N—H⋯O hydrogen bonds of the ammonium groups atoms to sulfate anions, a three-dimensional framework is formed (Table 1 ▶, Fig. 2 ▶).

## Synthesis and crystallization   

The title compound was obtained by reaction of an aqueous solution of praseodymium(III) sulfate with ethyl­enedi­amine and sulfuric acid (18 mol/l) in a stoichiometric ratio 1:1:2. The title compound crystallized by slow evaporation of the solvent at room temperature in form of light-green crystals with dimensions up to 3 mm within a few weeks.

## Refinement   

Crystal data, data collection and structure refinement details are summarized in Table 2 ▶. All H atoms were clearly discernible from difference Fourier maps. Methyl­ene H atoms were refined with a riding-model constraint, using a C—H distance of 0.97 Å and *U*_iso_(H) = 1.2*U*_eq_(C). Ammonium and water H atoms were refined freely.

## Supplementary Material

Crystal structure: contains datablock(s) I, global. DOI: 10.1107/S1600536814020704/wm5057sup1.cif

Structure factors: contains datablock(s) I. DOI: 10.1107/S1600536814020704/wm5057Isup2.hkl

CCDC reference: 1024418

Additional supporting information:  crystallographic information; 3D view; checkCIF report

## Figures and Tables

**Table 1:** Hydrogen-bond geometry (Å, °)

*D*—H⋯*A*	*D*—H	H⋯*A*	*D*⋯*A*	*D*—H⋯*A*
O1—H11⋯O32	0.72 (8)	2.53 (8)	2.974 (6)	121 (7)
O1—H12⋯O13	0.78 (6)	1.92 (6)	2.674 (5)	162 (6)
O2—H21⋯O3	1.00 (12)	1.89 (12)	2.858 (7)	163 (9)
O2—H22⋯O21^i^	0.77 (8)	2.29 (8)	2.905 (6)	137 (7)
O3—H31⋯O11^ii^	0.87 (7)	1.95 (8)	2.795 (5)	165 (7)
O3—H32⋯O12^iii^	0.80 (8)	2.00 (8)	2.766 (5)	162 (8)
N1—H1*A*⋯O33	0.87 (8)	2.48 (8)	3.291 (5)	155 (6)
N1—H1*B*⋯O3	0.88 (7)	1.92 (7)	2.758 (6)	158 (6)
N1—H1*C*⋯O13^iv^	0.99 (9)	1.85 (9)	2.841 (6)	176 (7)
N2—H2*A*⋯O24	0.76 (7)	2.21 (7)	2.976 (5)	177 (7)
N2—H2*B*⋯O22^v^	0.83 (8)	2.17 (8)	2.967 (6)	162 (7)
N2—H2*C*⋯O34^vi^	0.94 (7)	2.20 (6)	3.020 (5)	146 (5)
N3—H3*A*⋯O2^vii^	0.85 (7)	2.12 (7)	2.901 (8)	153 (6)
N3—H3*B*⋯O11	0.90 (7)	1.95 (8)	2.847 (6)	175 (6)
N3—H3*C*⋯O33	0.87 (7)	2.20 (7)	3.066 (5)	173 (6)

Symmetry codes: (i) 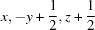; (ii) 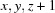; (iii) 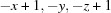; (iv) 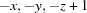; (v) 
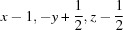
; (vi) 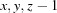; (vii) 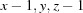.

**Table 2:** Experimental details

Crystal data	
Chemical formula	(C_2_H_10_N_2_)_3_[Pr_2_(SO_4_)_6_]·6H_2_O
*M*_r_	1152.70
Crystal system, space group	Monoclinic, *P*2_1_/*c*
Temperature (K)	295
*a*, *b*, *c* (Å)	6.6174 (8), 26.668 (4), 10.0264 (13)
β (°)	104.446 (15)
*V* (Å^3^)	1713.4 (4)
*Z*	2
Radiation type	Mo *K*α
μ (mm^−1^)	3.29
Crystal size (mm)	0.22 × 0.21 × 0.20

Data collection	
Diffractometer	Stoe *IPDS*-II
Absorption correction	Multi-scan (*X-SHAPE* and *X-RED32*; Stoe & Cie, 2002 ▶)
*T*_min_, *T*_max_	0.491, 0.620
No. of measured, independent and observed [*I* > 2σ(*I*)] reflections	14346, 3922, 3091
*R*_int_	0.044
(sin θ/λ)_max_ (Å^−1^)	0.662

Refinement	
*R*[*F* ^2^ > 2σ(*F* ^2^)], *wR*(*F* ^2^), *S*	0.028, 0.069, 0.97
No. of reflections	3923
No. of parameters	311
H-atom treatment	All H-atom parameters refined
Δρ_max_, Δρ_min_ (e Å^−3^)	0.72, −1.08

Computer programs: *X-AREA* (Stoe & Cie, 2002 ▶), *SIR92* (Altomare *et al.*, 1993 ▶), *SHELXL97* (Sheldrick, 2008 ▶), *ATOMS* (Dowty, 2002 ▶) and *publCIF* (Westrip, 2010 ▶).

## supplementary crystallographic information

### Crystal data

**Table:**

(C_2_H_10_N_2_)_3_[Pr_2_(SO_4_)_6_]·6H_2_O	*F*(000) = 1148
*M**_r_* = 1152.70	*D*_x_ = 2.234 Mg m^−^^3^
Monoclinic, *P*2_1_/*c*	Mo *K*α radiation, λ = 0.71073 Å
Hall symbol: -P 2ybc	Cell parameters from 25 reflections
*a* = 6.6174 (8) Å	θ = 20.0–24.3°
*b* = 26.668 (4) Å	µ = 3.29 mm^−^^1^
*c* = 10.0264 (13) Å	*T* = 295 K
β = 104.446 (15)°	Parallelepiped, light-green
*V* = 1713.4 (4) Å^3^	0.22 × 0.21 × 0.20 mm
*Z* = 2	

### Data collection

**Table:**

Stoe IPDS-II diffractometer	3922 independent reflections
Radiation source: fine-focus sealed tube	3091 reflections with *I* > 2σ(*I*)
Graphite monochromator	*R*_int_ = 0.044
ω and φ scans	θ_max_ = 28.1°, θ_min_ = 2.6°
Absorption correction: multi-scan (*X-SHAPE* and *X-RED32*; Stoe & Cie, 2002)	*h* = −8→8
*T*_min_ = 0.491, *T*_max_ = 0.620	*k* = −34→35
14346 measured reflections	*l* = −13→13

### Refinement

**Table:**

Refinement on *F*^2^	Secondary atom site location: difference Fourier map
Least-squares matrix: full	Hydrogen site location: inferred from neighbouring sites
*R*[*F*^2^ > 2σ(*F*^2^)] = 0.028	All H-atom parameters refined
*wR*(*F*^2^) = 0.069	*w* = 1/[σ^2^(*F*_o_^2^) + (0.0454*P*)^2^] where *P* = (*F*_o_^2^ + 2*F*_c_^2^)/3
*S* = 0.97	(Δ/σ)_max_ < 0.001
3923 reflections	Δρ_max_ = 0.72 e Å^−^^3^
311 parameters	Δρ_min_ = −1.08 e Å^−^^3^
0 restraints	Extinction correction: *SHELXL*, Fc^*^=kFc[1+0.001xFc^2^λ^3^/sin(2θ)]^-1/4^
Primary atom site location: structure-invariant direct methods	Extinction coefficient: 0.0150 (6)

### Special details

**Table:**

Experimental. A suitable single-crystal was carefully selected under a polarizing microscope and mounted in a glass capillary. The scattering intensities were collected on an imaging plate diffractometer (*IPDS* II, Stoe & Cie) equipped with a fine focus sealed tube X-ray source (Mo Kα, λ = 0.71073 Å) operating at 50 kV and 30 mA. Intensity data for the title compound were collected at room temperature by ω-scans in 180 frames (0 < ω < 180°; φ = 0° and 90°, Δω = 2°, exposure time of 10 min) in the 2Θ range 2.29 to 59.53°. Structure solution and refinement were carried out using the programs *SIR97* (Altomare *et al.*, 1999) and *SHELXL97* (Sheldrick, 2008). The last cycles of refinement included atomic positions and anisotropic parameters for all atoms. The final difference maps were free of any chemically significant features. The refinement was based on F^2^ for ALL reflections.
Geometry. All e.s.d.'s (except the e.s.d. in the dihedral angle between two l.s. planes) are estimated using the full covariance matrix. The cell e.s.d.'s are taken into account individually in the estimation of e.s.d.'s in distances, angles and torsion angles; correlations between e.s.d.'s in cell parameters are only used when they are defined by crystal symmetry. An approximate (isotropic) treatment of cell e.s.d.'s is used for estimating e.s.d.'s involving l.s. planes.
Refinement. Refinement of *F*^2^ against ALL reflections. The weighted *R*-factor *wR* and goodness of fit *S* are based on *F*^2^, conventional *R*-factors *R* are based on *F*, with *F* set to zero for negative *F*^2^. The threshold expression of *F*^2^ > σ(*F*^2^) is used only for calculating *R*-factors(gt) *etc*. and is not relevant to the choice of reflections for refinement. *R*-factors based on *F*^2^ are statistically about twice as large as those based on *F*, and *R*- factors based on ALL data will be even larger.

### Fractional atomic coordinates and isotropic or equivalent isotropic displacement parameters (Å^2^)

**Table:**

	*x*	*y*	*z*	*U*_iso_*/*U*_eq_	
Pr	0.54622 (3)	0.177147 (7)	0.465046 (19)	0.01075 (8)	
S1	0.40656 (17)	0.06427 (3)	0.24648 (10)	0.0167 (2)	
S2	0.52984 (15)	0.25100 (3)	0.21431 (9)	0.01307 (18)	
S3	0.03251 (16)	0.15704 (4)	0.54283 (9)	0.0158 (2)	
O11	0.1979 (5)	0.05421 (12)	0.1565 (3)	0.0283 (7)	
O12	0.5682 (6)	0.05230 (12)	0.1764 (3)	0.0294 (7)	
O13	0.4331 (5)	0.03414 (11)	0.3740 (3)	0.0269 (7)	
O14	0.4203 (5)	0.11866 (10)	0.2836 (3)	0.0259 (7)	
O21	0.5603 (5)	0.30594 (11)	0.2203 (3)	0.0205 (6)	
O22	0.7151 (5)	0.22494 (11)	0.3007 (3)	0.0205 (6)	
O23	0.3524 (5)	0.23557 (10)	0.2694 (3)	0.0183 (6)	
O24	0.4972 (5)	0.23684 (10)	0.0667 (3)	0.0177 (6)	
O31	0.1767 (5)	0.17700 (11)	0.4653 (3)	0.0222 (6)	
O32	0.1426 (6)	0.12685 (14)	0.6597 (4)	0.0382 (9)	
O33	−0.1327 (5)	0.12693 (11)	0.4480 (3)	0.0235 (6)	
O34	−0.0807 (5)	0.19921 (12)	0.5890 (3)	0.0227 (6)	
O1	0.5360 (6)	0.09619 (12)	0.5899 (4)	0.0298 (8)	
H11	0.505 (12)	0.097 (3)	0.654 (8)	0.06 (2)*	
H12	0.501 (9)	0.074 (2)	0.539 (6)	0.030 (15)*	
O2	0.5314 (10)	0.1112 (3)	0.9028 (7)	0.100 (3)	
H21	0.438 (17)	0.082 (4)	0.883 (10)	0.12 (4)*	
H22	0.495 (12)	0.138 (3)	0.877 (8)	0.06 (2)*	
O3	0.2361 (7)	0.03214 (16)	0.8914 (4)	0.0380 (9)	
H31	0.220 (11)	0.033 (3)	0.975 (8)	0.056 (19)*	
H32	0.310 (13)	0.009 (3)	0.888 (8)	0.07 (3)*	
N1	−0.0784 (7)	0.02857 (16)	0.6520 (5)	0.0283 (8)	
H1A	−0.090 (11)	0.060 (3)	0.623 (7)	0.06 (2)*	
H1B	−0.005 (11)	0.032 (3)	0.738 (7)	0.053 (19)*	
H1C	−0.204 (14)	0.008 (3)	0.646 (9)	0.08 (3)*	
C1	0.0524 (8)	0.0008 (2)	0.5757 (5)	0.0300 (10)	
H1D	0.082 (10)	−0.034 (3)	0.615 (6)	0.048 (17)*	
H1E	0.173 (11)	0.014 (2)	0.595 (6)	0.045 (18)*	
N2	0.0775 (8)	0.20727 (16)	−0.1020 (4)	0.0257 (8)	
H2A	0.183 (11)	0.216 (2)	−0.057 (7)	0.040 (18)*	
H2B	−0.011 (12)	0.230 (3)	−0.113 (7)	0.06 (2)*	
H2C	0.086 (10)	0.199 (2)	−0.192 (7)	0.042 (16)*	
C2	0.0133 (9)	0.16060 (18)	−0.0433 (5)	0.0278 (10)	
H2D	0.115 (10)	0.136 (2)	−0.031 (6)	0.044 (17)*	
H2E	−0.095 (13)	0.144 (3)	−0.107 (8)	0.08 (3)*	
N3	−0.1249 (8)	0.12597 (16)	0.1435 (5)	0.0279 (9)	
H3A	−0.245 (11)	0.119 (2)	0.094 (6)	0.039 (17)*	
H3B	−0.023 (12)	0.103 (3)	0.153 (7)	0.06 (2)*	
H3C	−0.126 (11)	0.129 (3)	0.230 (8)	0.05 (2)*	
C3	−0.0508 (8)	0.17187 (17)	0.0867 (5)	0.0257 (9)	
H3D	0.054 (10)	0.184 (2)	0.148 (6)	0.031 (15)*	
H3E	−0.145 (9)	0.197 (2)	0.085 (6)	0.035 (15)*	

### Atomic displacement parameters (Å^2^)

**Table:**

	*U*^11^	*U*^22^	*U*^33^	*U*^12^	*U*^13^	*U*^23^
Pr	0.01069 (13)	0.01111 (11)	0.01082 (11)	0.00001 (7)	0.00336 (7)	−0.00053 (8)
S1	0.0203 (6)	0.0136 (4)	0.0153 (4)	−0.0014 (3)	0.0026 (3)	−0.0027 (3)
S2	0.0144 (5)	0.0144 (4)	0.0107 (4)	0.0007 (3)	0.0038 (3)	0.0022 (3)
S3	0.0113 (5)	0.0182 (4)	0.0184 (4)	0.0002 (3)	0.0046 (3)	0.0041 (4)
O11	0.0219 (18)	0.0306 (17)	0.0283 (16)	−0.0023 (13)	−0.0014 (13)	−0.0069 (13)
O12	0.032 (2)	0.0285 (17)	0.0332 (17)	0.0017 (13)	0.0178 (14)	−0.0027 (13)
O13	0.037 (2)	0.0216 (15)	0.0219 (15)	−0.0055 (13)	0.0063 (13)	0.0041 (12)
O14	0.038 (2)	0.0132 (14)	0.0225 (15)	−0.0009 (12)	0.0005 (13)	−0.0048 (11)
O21	0.0296 (18)	0.0156 (13)	0.0170 (13)	−0.0024 (11)	0.0070 (12)	−0.0003 (10)
O22	0.0148 (16)	0.0265 (15)	0.0203 (14)	0.0032 (11)	0.0045 (11)	0.0095 (11)
O23	0.0148 (16)	0.0227 (14)	0.0192 (13)	−0.0006 (11)	0.0075 (11)	0.0064 (11)
O24	0.0223 (16)	0.0190 (14)	0.0128 (13)	0.0025 (11)	0.0064 (11)	0.0012 (10)
O31	0.0144 (16)	0.0258 (15)	0.0290 (15)	−0.0014 (11)	0.0101 (11)	0.0065 (12)
O32	0.0218 (19)	0.049 (2)	0.042 (2)	0.0036 (15)	0.0032 (15)	0.0301 (17)
O33	0.0167 (17)	0.0166 (14)	0.0376 (17)	−0.0033 (11)	0.0076 (12)	−0.0072 (12)
O34	0.0159 (17)	0.0282 (16)	0.0243 (15)	−0.0017 (12)	0.0057 (12)	−0.0115 (12)
O1	0.050 (2)	0.0174 (16)	0.0246 (17)	−0.0035 (14)	0.0150 (16)	−0.0012 (14)
O2	0.067 (4)	0.106 (5)	0.101 (5)	−0.043 (4)	−0.026 (3)	0.074 (4)
O3	0.049 (3)	0.039 (2)	0.0219 (17)	0.0071 (18)	0.0004 (15)	−0.0071 (15)
N1	0.030 (2)	0.023 (2)	0.033 (2)	−0.0027 (16)	0.0083 (17)	−0.0027 (17)
C1	0.023 (3)	0.037 (3)	0.028 (2)	0.001 (2)	0.0030 (19)	−0.004 (2)
N2	0.026 (3)	0.025 (2)	0.026 (2)	−0.0076 (17)	0.0056 (17)	0.0038 (16)
C2	0.036 (3)	0.023 (2)	0.030 (2)	0.0009 (19)	0.020 (2)	0.0038 (18)
N3	0.026 (3)	0.033 (2)	0.028 (2)	−0.0022 (17)	0.0126 (18)	0.0050 (17)
C3	0.028 (3)	0.022 (2)	0.030 (2)	−0.0035 (18)	0.0127 (19)	0.0000 (18)

### Geometric parameters (Å, º)

**Table:**

Pr—O14	2.383 (3)	O34—Pr^iv^	2.541 (3)
Pr—O31	2.446 (3)	O1—H11	0.72 (8)
Pr—O1	2.505 (3)	O1—H12	0.78 (6)
Pr—O34^i^	2.541 (3)	O2—H21	1.00 (12)
Pr—O22	2.551 (3)	O2—H22	0.77 (8)
Pr—O33^i^	2.553 (3)	O3—H31	0.87 (7)
Pr—O24^ii^	2.564 (3)	O3—H32	0.80 (8)
Pr—O21^ii^	2.577 (3)	N1—C1	1.487 (6)
Pr—O23	2.582 (3)	N1—H1A	0.87 (8)
Pr—S3^i^	3.1621 (11)	N1—H1B	0.88 (7)
Pr—S2^ii^	3.1725 (9)	N1—H1C	0.99 (9)
Pr—S2	3.1741 (9)	C1—C1^v^	1.504 (9)
S1—O12	1.454 (3)	C1—H1D	1.01 (7)
S1—O11	1.473 (3)	C1—H1E	0.84 (7)
S1—O13	1.483 (3)	N2—C2	1.483 (6)
S1—O14	1.495 (3)	N2—H2A	0.76 (7)
S2—O23	1.475 (3)	N2—H2B	0.83 (8)
S2—O21	1.478 (3)	N2—H2C	0.94 (7)
S2—O22	1.485 (3)	C2—C3	1.499 (7)
S2—O24	1.490 (3)	C2—H2D	0.92 (7)
S3—O32	1.458 (3)	C2—H2E	0.94 (8)
S3—O31	1.472 (3)	N3—C3	1.484 (6)
S3—O34	1.488 (3)	N3—H3A	0.85 (7)
S3—O33	1.492 (3)	N3—H3B	0.90 (7)
O21—Pr^iii^	2.577 (3)	N3—H3C	0.87 (7)
O24—Pr^iii^	2.564 (3)	C3—H3D	0.87 (6)
O33—Pr^iv^	2.553 (3)	C3—H3E	0.92 (6)
			
O14—Pr—O31	80.82 (11)	O31—S3—O33	109.05 (18)
O14—Pr—O1	76.67 (11)	O34—S3—O33	105.04 (17)
O31—Pr—O1	81.17 (12)	S1—O14—Pr	144.42 (18)
O14—Pr—O34^i^	129.57 (10)	S2—O21—Pr^iii^	99.34 (13)
O31—Pr—O34^i^	148.16 (10)	S2—O22—Pr	100.34 (14)
O1—Pr—O34^i^	95.70 (12)	S2—O23—Pr	99.30 (14)
O14—Pr—O22	87.71 (10)	S2—O24—Pr^iii^	99.60 (14)
O31—Pr—O22	126.92 (9)	S3—O31—Pr	141.33 (18)
O1—Pr—O22	145.50 (11)	S3—O33—Pr^iv^	99.49 (14)
O34^i^—Pr—O22	70.82 (9)	S3—O34—Pr^iv^	100.13 (14)
O14—Pr—O33^i^	75.16 (10)	Pr—O1—H11	117 (6)
O31—Pr—O33^i^	148.01 (9)	Pr—O1—H12	112 (4)
O1—Pr—O33^i^	73.03 (12)	H11—O1—H12	121 (7)
O34^i^—Pr—O33^i^	55.31 (9)	H21—O2—H22	122 (8)
O22—Pr—O33^i^	73.30 (10)	H31—O3—H32	107 (7)
O14—Pr—O24^ii^	146.56 (10)	C1—N1—H1A	108 (5)
O31—Pr—O24^ii^	77.02 (10)	C1—N1—H1B	108 (5)
O1—Pr—O24^ii^	123.41 (11)	H1A—N1—H1B	102 (6)
O34^i^—Pr—O24^ii^	78.58 (9)	C1—N1—H1C	106 (5)
O22—Pr—O24^ii^	85.94 (9)	H1A—N1—H1C	120 (7)
O33^i^—Pr—O24^ii^	133.31 (9)	H1B—N1—H1C	111 (6)
O14—Pr—O21^ii^	142.34 (10)	N1—C1—C1^v^	110.7 (5)
O31—Pr—O21^ii^	77.75 (10)	N1—C1—H1D	110 (4)
O1—Pr—O21^ii^	69.73 (11)	C1^v^—C1—H1D	111 (4)
O34^i^—Pr—O21^ii^	71.56 (10)	N1—C1—H1E	109 (4)
O22—Pr—O21^ii^	129.86 (10)	C1^v^—C1—H1E	114 (4)
O33^i^—Pr—O21^ii^	109.50 (10)	H1D—C1—H1E	102 (5)
O24^ii^—Pr—O21^ii^	54.92 (8)	C2—N2—H2A	109 (5)
O14—Pr—O23	78.58 (10)	C2—N2—H2B	114 (5)
O31—Pr—O23	72.22 (9)	H2A—N2—H2B	112 (7)
O1—Pr—O23	146.05 (12)	C2—N2—H2C	106 (4)
O34^i^—Pr—O23	118.03 (10)	H2A—N2—H2C	112 (6)
O22—Pr—O23	54.71 (9)	H2B—N2—H2C	104 (6)
O33^i^—Pr—O23	121.97 (10)	N2—C2—C3	110.4 (4)
O24^ii^—Pr—O23	70.99 (9)	N2—C2—H2D	112 (4)
O21^ii^—Pr—O23	122.48 (9)	C3—C2—H2D	112 (4)
O12—S1—O11	110.7 (2)	N2—C2—H2E	112 (5)
O12—S1—O13	110.8 (2)	C3—C2—H2E	110 (5)
O11—S1—O13	108.69 (19)	H2D—C2—H2E	100 (6)
O12—S1—O14	109.09 (19)	C3—N3—H3A	108 (4)
O11—S1—O14	108.60 (19)	C3—N3—H3B	107 (5)
O13—S1—O14	108.92 (17)	H3A—N3—H3B	118 (6)
O23—S2—O21	111.99 (17)	C3—N3—H3C	113 (5)
O23—S2—O22	105.65 (16)	H3A—N3—H3C	111 (6)
O21—S2—O22	110.84 (18)	H3B—N3—H3C	99 (6)
O23—S2—O24	111.67 (17)	N3—C3—C2	111.2 (4)
O21—S2—O24	106.03 (16)	N3—C3—H3D	109 (4)
O22—S2—O24	110.75 (17)	C2—C3—H3D	110 (4)
O32—S3—O31	111.4 (2)	N3—C3—H3E	109 (4)
O32—S3—O34	110.9 (2)	C2—C3—H3E	118 (4)
O31—S3—O34	109.50 (18)	H3D—C3—H3E	99 (5)
O32—S3—O33	110.8 (2)		

Symmetry codes: (i) *x*+1, *y*, *z*; (ii) *x*, −*y*+1/2, *z*+1/2; (iii) *x*, −*y*+1/2, *z*−1/2; (iv) *x*−1, *y*, *z*; (v) −*x*, −*y*, −*z*+1.

### Hydrogen-bond geometry (Å, º)

**Table:**

*D*—H···*A*	*D*—H	H···*A*	*D*···*A*	*D*—H···*A*
O1—H11···O32	0.72 (8)	2.53 (8)	2.974 (6)	121 (7)
O1—H12···O13	0.78 (6)	1.92 (6)	2.674 (5)	162 (6)
O2—H21···O3	1.00 (12)	1.89 (12)	2.858 (7)	163 (9)
O2—H22···O21^ii^	0.77 (8)	2.29 (8)	2.905 (6)	137 (7)
O3—H31···O11^vi^	0.87 (7)	1.95 (8)	2.795 (5)	165 (7)
O3—H32···O12^vii^	0.80 (8)	2.00 (8)	2.766 (5)	162 (8)
N1—H1*A*···O33	0.87 (8)	2.48 (8)	3.291 (5)	155 (6)
N1—H1*B*···O3	0.88 (7)	1.92 (7)	2.758 (6)	158 (6)
N1—H1*C*···O13^v^	0.99 (9)	1.85 (9)	2.841 (6)	176 (7)
N2—H2*A*···O24	0.76 (7)	2.21 (7)	2.976 (5)	177 (7)
N2—H2*B*···O22^viii^	0.83 (8)	2.17 (8)	2.967 (6)	162 (7)
N2—H2*C*···O34^ix^	0.94 (7)	2.20 (6)	3.020 (5)	146 (5)
N3—H3*A*···O2^x^	0.85 (7)	2.12 (7)	2.901 (8)	153 (6)
N3—H3*B*···O11	0.90 (7)	1.95 (8)	2.847 (6)	175 (6)
N3—H3*C*···O33	0.87 (7)	2.20 (7)	3.066 (5)	173 (6)

Symmetry codes: (ii) *x*, −*y*+1/2, *z*+1/2; (v) −*x*, −*y*, −*z*+1; (vi) *x*, *y*, *z*+1; (vii) −*x*+1, −*y*, −*z*+1; (viii) *x*−1, −*y*+1/2, *z*−1/2; (ix) *x*, *y*, *z*−1; (x) *x*−1, *y*, *z*−1.
